# Effect of papain-based gel on type I collagen - spectroscopy applied for microstructural analysis

**DOI:** 10.1038/srep11448

**Published:** 2015-06-23

**Authors:** Zenildo Santos Silva Júnior, Sergio Brossi Botta, Patricia Aparecida Ana, Cristiane Miranda França, Kristianne Porta Santos Fernandes, Raquel Agnelli Mesquita-Ferrari, Alessandro Deana, Sandra Kalil Bussadori

**Affiliations:** 1 Postgraduate Program in Biophotonics Applied to Health Sciences, Nove de Julho University (UNINOVE), São Paulo, SP, Brazil; 2 School of Dentistry, Nove de Julho University, São Paulo, SP, Brazil; 3 Biomedical Engineering, Center of Engineering, Modeling and Applied Social Sciences, Federal University of ABC, Sao Bernardo do Campo, SP, Brazil

## Abstract

Considering the improvement of biomaterials that facilitate atraumatic restorative techniques in dentistry, a papain-based gel can be used in the chemomechanical removal of decayed dental tissue. However, there is no information regarding the influence of this gel on the structure of sound collagen. The aim of the present study was to investigate the adsorption of a papain-based gel (Papacarie^TM^) to collagen and determine collagen integrity after treatment. A pilot study was first performed with 10 samples of type I collagen membrane obtained from bovine Achilles deep tendon to compare the influence of hydration (Milli-Q water) on infrared bands of collagen. In a further experiment, 10 samples of type I collagen membrane were used to evaluate the effects of Papacarie^TM^ on the collagen microstructure. All analyses were performed using the attenuated total reflectance technique of Fourier transform infrared (ATR-FTIR). The results demonstrated that the application of Papacarie^TM^ does not lead to the degradation of collagen and this product can be safely used in minimally invasive dentistry. As the integrity of sound collagen is preserved after the application of the papain-based gel, this product is indicated for the selective removal of infected dentin, leaving the affected dentin intact and capable of re-mineralization.

In dentistry, minimally invasive treatment is based on the selective removal of decayed tissue, which has led to a transformation in the paradigm of restorative treatment for tooth decay, allowing the maximum preservation of sound tooth structure and tissue capable of re-mineralization^1^,^2^. According to the literature, an active carious lesion on dentin is divided into four layers^1^,^2^,^3^,^4^. The necrotic surface layer and underlying infected dentin, which has a large number of bacteria, is completely de-mineralized, has a disorganized collagen structure and must be removed. The affected layer immediately below the infected layer exhibits little change in the three-dimensional structure of collagen, has few or no bacteria, is capable of re-mineralization and should be preserved. The deepest layer of dentin is more mineralized, has obliterated dentinal tubules and intact collagen and should also be preserved^5^.

The aim of the partial removal of carious lesions in minimally invasive techniques is to leave the affected dentin, which allows the preservation and re-mineralization of the dental structure and avoids pulp exposure^6^,^7^. Chemomechanical agents can be used for this purpose. Such techniques are capable of removing only infected, necrotic tissue and ensure the preservation of the non-infected layers. Despite the advantages of chemomechanical methods for the partial removal of dentinal caries, only the pupal wall can have affected dentin. All other walls (axial walls and dentino-enamel junction surfaces) must be checked for complete caries removal.

The restoration of cavities using minimally invasive methods requires materials such as composite resins or glass ionomer cement, which bond to the dentinal surface. Thus, the treatment of the dentinal surface and the resulting characteristics of the dentinal substrate are important to adhesion and affect the performance of composite resin restorations, as adhesion is mainly based on micromechanical retention by the formation of a hybrid layer and resin tags within the dentinal tubules.

For the restoration of cavities with affected, partially demineralized dentin on the pulpal wall, phosphoric acid or acid primers are not recommended, as such products can lead to further demineralization. Collagen on the surrounding walls must be intact for the proper adhesion of composite resin restorations. Thus, the application of chemomechanical methods, such as papain-based gel, on the sound surrounding walls should not alter the mechanical properties of collagen fibers.

Despite their common use, direct dental composite resin systems have short durability (approximately 5 to 8 years). Therefore, a stable bond between the adhesive system and collagen fibers forming the hybrid layer remains a challenge in restorative dentistry.

As the durability of the bond between the dentin and adhesive system depends on the structural stability and mechanical properties of collagen fibers, attempts to maintain these fibers non-denatured could help the bond. The mechanical properties of collagen are of extreme importance to the formation of the hybrid layer. Thus, glass ionomer cement should be used on the pulpal wall to stimulate re-mineralization and composite resin should be used to restore the cavity.

An enzyme-based gel denominated Papacarie^TM^ is one of the agents employed for chemomechanical caries removal in minimally invasive treatment. This product was introduced in 2003 by Bussadori *et al*.^8^ and consists of a gel composed by papain, chloramine, toluidine blue, salts, preservatives, stabilizers, thickener and deionized water^2^,^9^,^10^,^11^,^12^. Papain is an enzyme similar to human pepsin and acts as an anti-inflammatory debriding agent that does no damage to sound tissue, accelerates the healing process and has bactericidal, bacteriostatic and anti-inflammatory properties^11^,^13^.

Carious dentin after the use of Papacarie^TM^ has no smear layer, likely due to the proteolytic nature of the papain gel, which removes necrotic and infected tissue composed of denatured collagen^12^. However, Bertassoni and Marshall^14^ showed that Papacarie^TM^ could partially degrade intact non-mineralized type I collagen fibrils from the tendon of rats, which could exert a negative influence on adhesion and the formation of the hybrid layer.

To clarify whether Papacarie^TM^ degrades intact collagen and alters the chemical structure of this protein, the aim of the present study was to evaluate the structure of type-I collagen under the action of the papain-based gel using the attenuated total reflectance technique of Fourier transform infrared (ATR-FTIR) spectroscopy.

## Materials and Methods

Sponges of bioabsorbable membranes made of type-I collagen from bovine Achilles deep tendon (Technodry Liofilizados Médicos, Brazil) were cut with a sterile biopsy punch to obtain discs measuring 5 mm in diameter and 2 mm in thickness. The discs were randomly distributed among the experimental groups (n = 5 per group).

In the pilot experiment, 10 samples of collagen (n = 5 per group) were used to determine the influence of water on the infrared bands. Two experimental groups were used in this pilot study (Table 1).

All samples were compositionally analyzed using ATR-FTIR spectroscopy with the aid of the Varian 610 spectrometer (Agilent Technologies, USA), with a DTGS detector using a diamond crystal. During the experiments, the samples were positioned individually over the diamond crystal on the ATR accessory and standardized pressure was applied to ensure optimal optical contact between the collagen sample and crystal as well as to keep the samples intact. A micrometric shifter was used to apply the same, slight pressure to all samples, which did not alter their characteristics or compromise the experimental results.

A single spectrum was collected for each sample, with the background spectra subtracted immediately before each acquisition. All spectra were obtained at a resolution of 4.0 cm^−1^, with 80 scans in the range of 4000 to 600 cm^−1^ 15, and were recorded using the Varian Resolutions Pro software program.

To evaluate the integrity of the collagen triple helix, peak absorbance ratios of 1235 cm^−1^/1450 cm^−1^ were considered. A ratio close to 0.5 denotes compromised integrity of the collagen triple helix, whereas a ratio closer to 1 denotes the maintenance of the integrity of amide III and the C-H bond of the pyrrolidine ring of the type I collagen triple helix^16^. The conversion of spectra and the analysis of collagen integrity were performed using the Origin Pro 8 (OriginLab Corp., USA) software program.

The peak heights were performed after a vetorial normalization of each spectrum using the Origin Pro 8 (OriginLab Corp., USA) software program.

After the pilot study, a further experiment was performed to evaluate the effects of Papacarie^TM^ on the collagen structure. For such, three experimental groups were considered (Table 2).

For G3, each sample was positioned in a plate and fully covered with the papain-based gel (Papacarie^TM^, F&A, Brazil). After 30 seconds, the samples were then carefully removed from the plate with sterile forceps (changed for each sample), abundantly washed with Milli-Q water (Millipore, Bedford, USA) for 30 s and lightly dried with filter paper (Whatman No. 6, Whatman International, England). The compositional analysis of all samples was performed immediately after treatments using ATR-FTIR spectroscopy.

### Statistical analysis

Levene’s test and the Shapiro-Wilk test were performed to determine the occurrence of equal variances and normality of the experimental data. The data were then statistically analyzed using one-way analysis of variance (ANOVA) followed by Tukey’s test (Minitab 14, Minitab Inc., USA) for pairwise comparisons between groups (α = 0.05). Power analysis is important to calculate the number of samples per experimental group needed to enable statistical judgments that are accurate and reliable. Performing the power analysis allows identifying significant differences. Power analysis (GPower, v.3.1.9.2) was performed to estimate the test power considering the sample size of each experimental group (n = 5), the results of one-way ANOVA and assuming a difference between means of α = 0.05 and β = 0.95. This analysis indicated a power between 0.997 and 0.999. For this experiment, it was necessary to calculate the effect size from a pilot study held previously. The calculated effect size value was 4.52763. The sampling size of five collagen membranes/treatment was established and confirmed by a pilot study, which demonstrated that three collagen membranes would be sufficient for a power of 95% (β = 0.05) to detect a difference among treatments at the 5% level (α = 0.05) in terms of collagen denaturing.

## Results

The collagen collagen peak assignments was based on Movasaghi *et al*.(2008)^17^.

**Figure 1:**
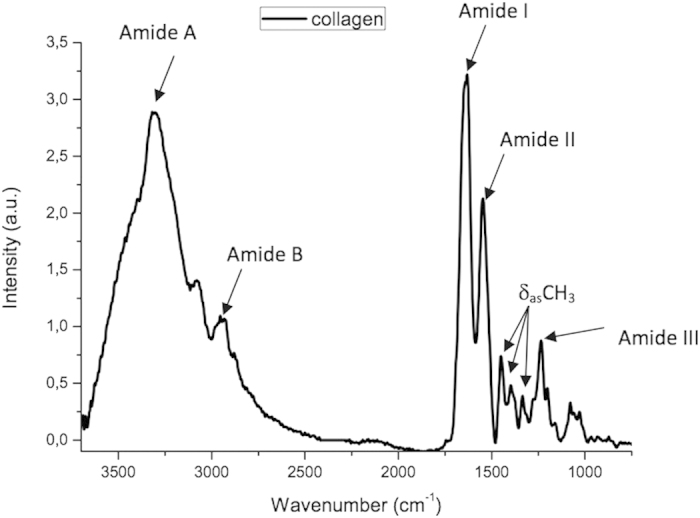
Mean infrared type I collagen spectrum obtained for pure (dry) membranes from bovine Achilles deep tendon in region of 3700 to 800 cm^−1^ (arrows indicate peaks of greater intensity).

Figure 1 displays the mean of infrared spectrum of the pure type I collagen membrane, in which it is possible to identify bands corresponding to amide A (3309 cm^−1^) and amide B (2930 cm^−1^) and the three main bands of the collagen fingerprint at 1630 cm^−1^ (typical of amide I due to carbonyl stretching – C-H), 1551 cm^−1^ (related to amide II due to vibrations on the plane of the N-H bond and C-N stretching) and 1232 cm^−1^ (corresponding to vibrations on the plane of amide III due to C-N stretching and N-H deformation). The peaks identified at 1454 cm^−1^ and in the region between 1417 cm^−1^ and 1360 cm^−1^ correspond to the stereochemistry of the pyrrolidine rings of proline and hydroxyproline. The peaks found in the region between 3100 cm^−1^ and 3400 cm^−1^ occur due to O-H and N-H stretching of amide A.

**Figure 2:**
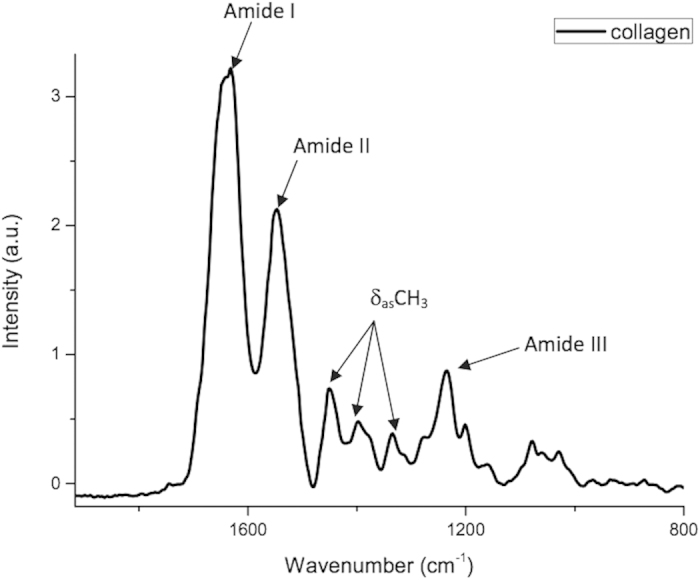
Mean infrared type I collagen spectrum obtained for pure (dry) membranes from bovine Achilles deep tendon in region of 1800 to 800 cm^−1^ (arrows indicate peaks of greater intensity in fingerprint region).

Figure 2 displays the region of the collagen fingerprint in greater detail, showing the main bands characteristic of collagen at 1630 cm^−1^ (amide I), 1551 cm^−1^ (amide II), 1232 cm^−1^ (amide III) and 1450 cm^−1^ (pyrrolidine rings of proline and hydroxyproline).

**Figure 3:**
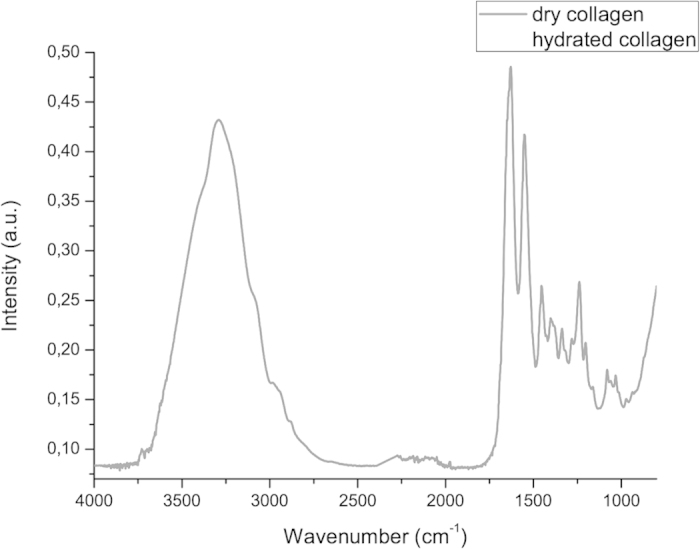
Mean infrared type I collagen spectrum obtained for dry membranes and membranes washed with Milli-Q water (hydrated) in region from 3800 to 800 cm^−1^.

Figure 3 displays the results of the pilot study. The exposure of the collagen membranes to Milli-Q water significantly increased the infrared band from 3000 to 3500 cm^−1^, which corresponds to asymmetric and symmetric O-H stretching. Moreover, an increase occurred in the intensity of all infrared bands in the fingerprint region in comparison to the dry collagen membranes.

The analysis of triple helix structure of dry and hydrated collagen demonstrated that the application of water did not affect the collagen structure, since the absorbance ratio from bands at 1235 cm^−1^ and 1450 cm^−1^ had mean values of 1.03 ± 0.01 and 1.00 ± 0.01 for dry and hydrated collagen, respectively.

**Figure 4:**
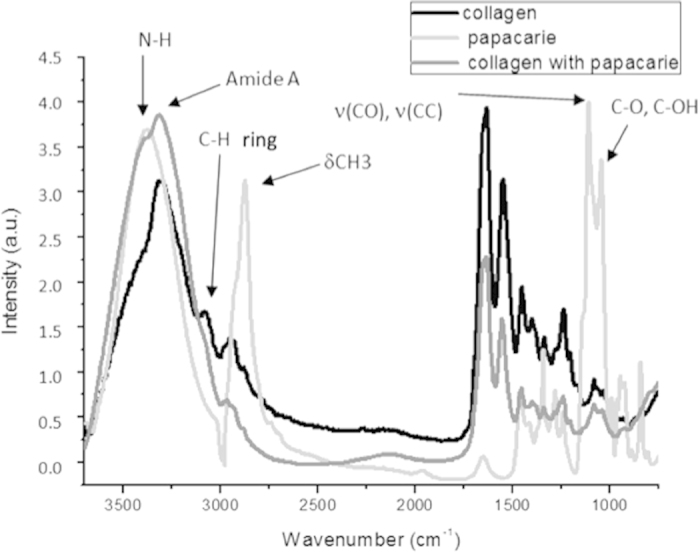
Mean infrared type I collagen spectrum obtained for hydrated type I collagen membrane, Papacarie^TM^ and type I collagen membranes treated with Papacarie^TM^ in region from 3800 to 800 cm^−1^ (arrows indicate main infrared absorption bands for Papacarie^TM^).

Figure 4 displays the mean infrared spectrum of Papacarie^TM^, showing corresponding infrared absorption bands of CO, COH, CC, NH and CH_3_. The figure also shows the mean infrared spectrum of collagen membranes treated with Papacarie^TM^, with increased absorbance of N-H and Amide A bands as well as a slight decrease in amide III absorbance. However, no new bands or the disappearance of bands were evident.

The analysis of the integrity of collagen triple helix mean absorbance ratio from bands at 1235 cm^−1^ (amide III) and 1450 cm^−1^ (pyrrolidine ring) showed that the application of PapacarieTM promoted statistically different (p = 0.01) between groups G1 (hydrated collagen group) which had a mean of 1.00 ± 0.01 and G3 (collagen treated with PapacarieTM) which had a mean of 0.884 ± 0.024. Otherwise, for both experimental groups, the ratios of mean absorbance at 1235 cm^−1^ and 1450 cm^−1^ were greater than 0.8, demonstrating a lack of collagen denaturation because according to Sylvester *et al*. (1989)^16^, values close to 0.5 denote a change in the three-dimensional structure of the type I collagen triple helix.

## Discussion

Papain gel can act as far as 100 um, as demonstrated by confocal laser scanning microscopy on dentin tissue^18^. As ATR-FTIR provides compositional analyses up to 10 um, the spectra in the present study were representative of treated collagen. The ATR-FTIR analysis of the integrity of the collagen triple helix demonstrated that Papacarie^TM^ does not cause significant collagen denaturation.

The literature states that the proteolytic enzyme papain in Papacarie^TM^ interacts with partially degraded collagen in the necrotic tissue of carious lesions, causing additional softening of this tissue^14^. This proteolytic action likely occurs only in the necrotic tissue, as sound tissue contains alpha-1-antitrypsin, which is an antiprotease that impedes the action of proteolytic enzymes^5^,^10^,^19^. In the present study, Papacarie^TM^ did not lead to the degradation of collagen, as demonstrated by FTIR values greater than 0.8 in all treatments. According to Sylvester *et al*. (1989)^16^, values close to 0.5 denote a change in the three-dimensional structure of the type I collagen triple helix. ATR-FTIR spectroscopy is highly indicated for the study of chemical changes produced in the collagen structure. Moreover, type I collagen fibers from human dentin are structurally similar to bovine Achilles deep tendon (employed in the present study) when evaluated using this imaging method^20^.

The infrared spectrum is characteristic of every molecule and certain groups of atoms give rise to bands that occur close to 1 and at the same frequency, irrespective of the structure of the molecule. It is precisely the presence of these characteristic bands of groups that allows the acquisition of useful structural information^15^. For different chemical groups, the wavelengths absorbed and the natural frequency of the vibrations are unique and depend on the existent bond type (C=C, C-H, C=O, N-H and O-H)^20^. The range with the greatest usefulness for the characterization of organic compounds is the mid infrared (4000 to 400 cm^−1^).

In the present study, the hydration of collagen sponges promoted an increase in all infrared peaks of collagen spectra (including the peaks corresponding to the collagen fingerprint) and with higher contribution of 3300 cm^−1^ and 1630 cm^−1^ peaks (Fig. 3). Thus, an increase in adsorbed water in collagen washed with Milli-Q water was found, as expected. As amide I has an infrared peak at 1630 cm^−1^, water adsorption contributed to the increase in the intensity of the amide I peak.

After the application of PapacarieTM gel (Fig. 4), an increase in adsorbed water was found, as evidenced by the increase at the 3300 cm^−1^ peak. This adsorbed water comes from the water that constitutes the gel of Papacarie^TM^. However, a decrease on the intensity of 1630 cm-1 peak was also found, which indicates a slight proteolytic action of Papacarie^TM^ gel, reducing the content of amide I. In this case, the effect of adsorbed water (which would increase the intensity of 1630 cm^−1^ peak) was not observed, but rather the action of papain on the structure of collagen was evident by the amide I peak, prevailing in relation to the water adsorption effect.

To calculate the integrity of the triple helix of collagen, the ratio of 1235 cm^−1^ (amide III) and 1450 cm^−1^ (pyrrolidine ring) bands was used, which are not affected by the water adsorption effect. Thus, it is possible to conclude that the amount of water in the Papacarie^TM^ gel did not interfere with its effects on the collagen structure.

In FTIR analysis, the intensities of amides I and II are related to the helical structure of collagen and the intensity of amide I could be used for characterizing the secondary structure of proteins^21^,^23^. Amides I and II are the main infrared absorption bands of the peptide group in collagen. Both bands absorb in different regions depending on whether or not they participate in hydrogen bonding interactions. Hydrogen bonds affect the vibration frequencies of the participating atoms. The presence of hydrogen bonds is an indication of polypeptide chains, assuming regular secondary structures. In fact, both amide I and II bands absorb in slightly different regions if they are in an A-like helix or in b-sheets^21^. Thus, amide bands can be used to probe the structure of collagen^23^.

The evaluation of the integrity of the collagen triple helix was performed by the analysis of the ratio of the absorbance of bands 1235 cm^−1^ (amide III) and 1450 cm^−1^ (stereochemistry of the pyrrolidine rings). Although the former is sensitive to the presence of the secondary structure of collagen, the latter is independent from the ordered structure of collagen^16^. For type I collagen fibers, the integrity of the secondary structure is demonstrated when the value of the 1235/1450 cm^−1^ ratio is close to 1. According to Sylvester *et al*. (1989)^16^, values close to 0.5 denote a change in the three-dimensional structure of the type I collagen triple helix

## Conclusion

The results of this study demonstrate the safety^14^,^23^ of Papacarie^TM^ as a selective caries removal agent. ATR-FTIR spectroscopy showed that this papain-based gel does not cause collagen denaturation, which demonstrates its safe use in minimally invasive dentistry, with the preservation of sound tooth structure and tissue capable of re-mineralization.

## Additional Information

**How to cite this article**: Júnior, Z. S. S. *et al*. Effect of papain-based gel on type I collagen - spectroscopy applied for microstructural analysis. *Sci. Rep*. **5**, 11448; doi: 10.1038/srep11448 (2015).

## Figures and Tables

**Table 1:**
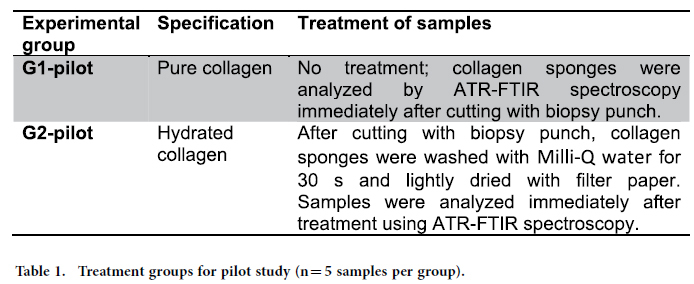
Treatment groups for pilot study (n = 5 samples per group).

**Table 2:**
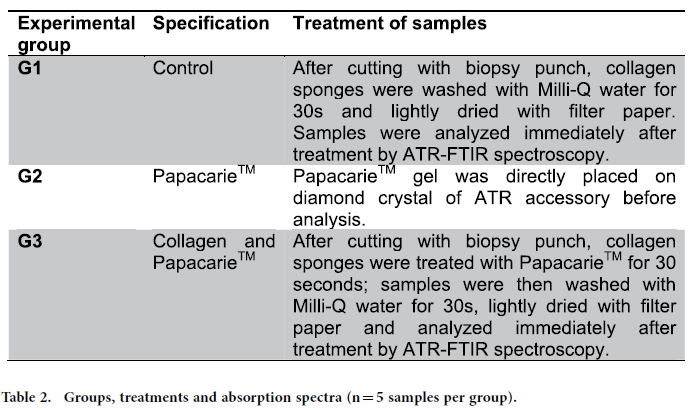
Groups, treatments and absorption spectra (n = 5 samples per group).
